# Buried penis and morbid obesity

**DOI:** 10.1007/s12024-022-00461-w

**Published:** 2022-02-23

**Authors:** Roger W. Byard, Luzern Tan

**Affiliations:** 1 Adelaide Medical School, The University of Adelaide, Frome Road, Adelaide, 5005 Australia; 2 Forensic Science South Australia, 21 Divett Place, Adelaide, South Australia 5005 Australia

**Keywords:** Buried penis, Morbid obesity, *Koro*, BMI, Autopsy assessment

## Abstract

Three morbidly obese men aged 69, 49 and 45 years with respective BMIs of 46.3, 49.1 and 59.3 died suddenly from underlying cardiovascular disease. At autopsy all were found to have marked penile shortening typical of an entity known as “buried penis.” This condition arises in adulthood most commonly from morbid obesity as the penile shaft becomes enveloped by encroaching suprapubic adipose tissue. It is associated with infective, obstructive and malignant complications. Histology will be required to identify less-common causative conditions or any inflammatory or premalignant/malignant changes.

## Case reports

A search was conducted of the case files of one of the authors (RWB) for males with morbid obesity (body mass index, BMI, > 40 kg/meters^2^) listed in the autopsy database at Forensic Science SA, Adelaide Australia over a 20-year period from 2002–2021. There were 56 cases with an age range of 25–79 years (mean 52.6 years) and a BMI range of 40.1–90.3 (mean 50.8). Three cases were recorded (5%) where there was a buried penis, i.e., where the otherwise normal penis had been almost completely enveloped by the suprapubic fat pad.

**Fig. 1:**
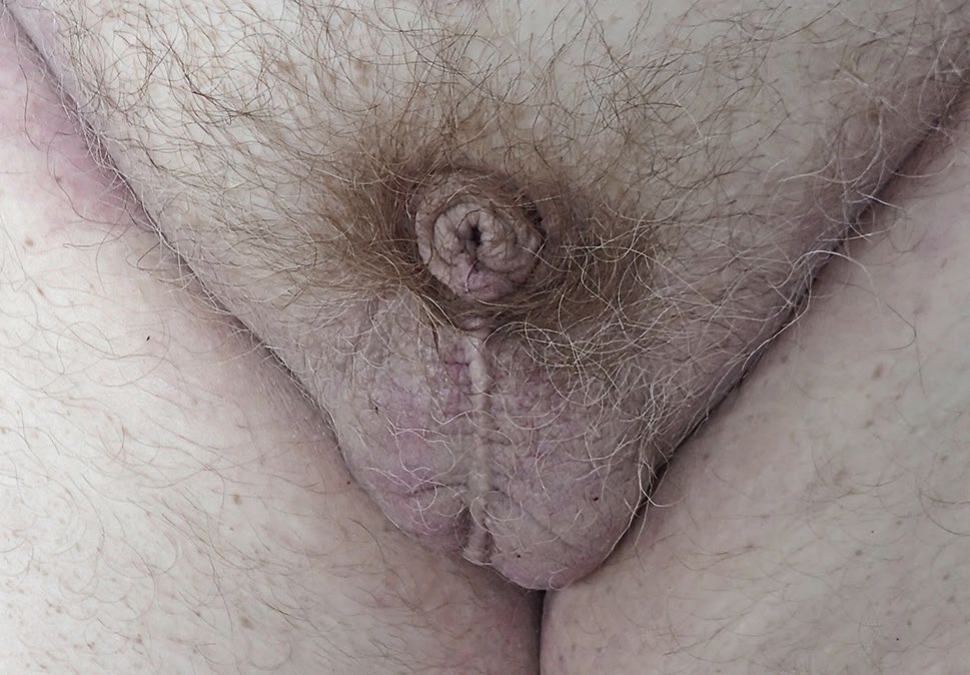
Genitalia of a 62-year-old morbidly obese man showing marked shortening of the penis with two testes in the scrotum

*Case 1*: A 62-year-old morbidly obese man (body mass index—BMI 46.3) complained of shortness of breath and then collapsed. He had a previous aortic valve replacement and a history of asthma. At autopsy, he had marked cardiomegaly with a heart weight of 1059 gms associated microscopically with both amyloid deposition and fibrous scarring. No myofibre disarray was present to suggest hypertrophic cardiomyopathy. There was no significant coronary artery atherosclerosis and no acute asthmatic changes, with the aortic valve replacement being in good order. Death was due to cardiomegaly with amyloid deposition complicating aortic valve replacement and morbid obesity. His genitalia showed marked penile shortening with two testes present in the scrotum (Fig. 1).

**Fig. 2:**
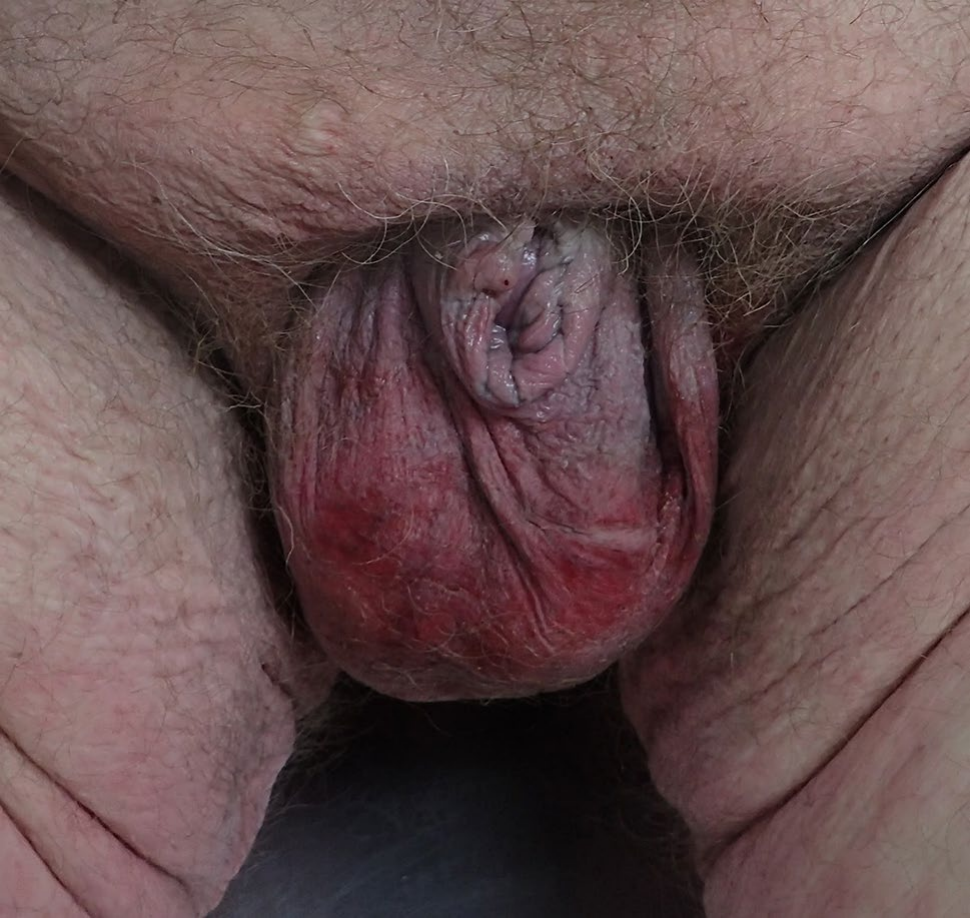
Genitalia of a 49-year-old morbidly obese man showing marked shortening of the penis with two testes in the scrotum beneath a prominent fatty apron

*Case 2*: A 49-year-old morbidly obese man (BMI 49.1 – with a right below knee amputation) collapsed at his home address. He had a past history of “multiple heart attacks,” hypertension, type 2 diabetes mellitus, a right leg amputation and two strokes. At autopsy, there was significant atherosclerotic narrowing of all three of the major epicardial coronary arteries associated with healed and healing myocardial infarcts and an acute myocardial infarction. The heart was also enlarged (611 g). Death was due to ischemic and hypertensive heart disease complicating morbid obesity. A prominent fatty apron was present with marked penile shortening and two testes present in the scrotum (Fig. 2).

**Fig. 3:**
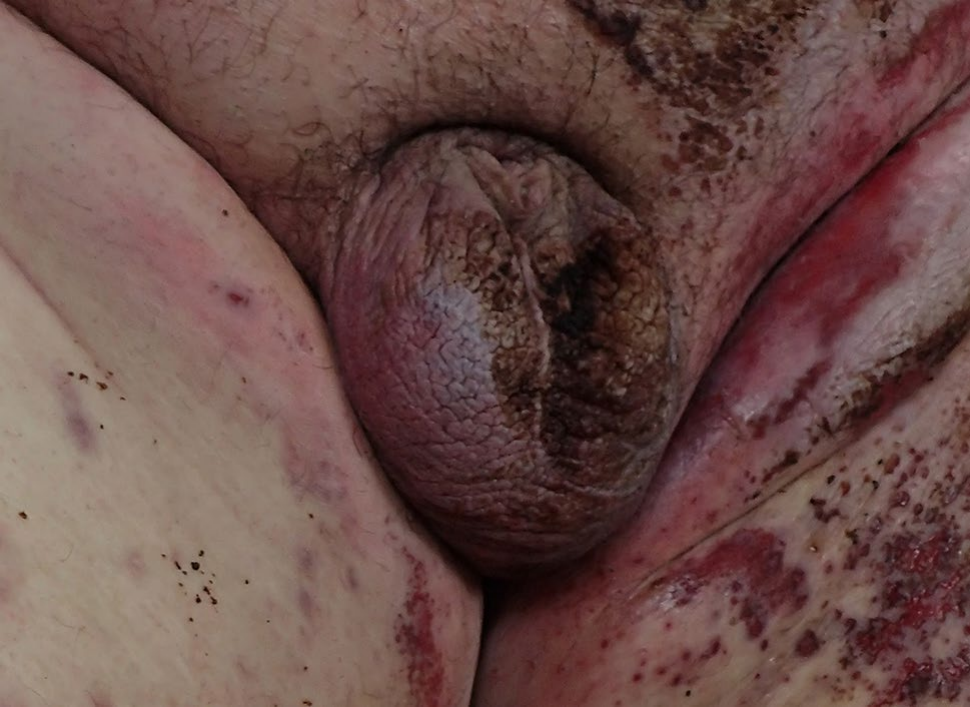
Genitalia of a 45-year-old morbidly obese man showing marked shortening of the penis with two testes in the scrotum. Fecal soiling and patchy postmortem parchmenting are also present

*Case 3*: A 45-year-old morbidly obese man (BMI 59.3) was found dead in his bed. Although he had no specific past medical history, he had been bed-bound for six to seven months. At autopsy, he had marked cardiomegaly with a heart weight of 1116 gms with no myofibre disarray to suggest hypertrophic cardiomyopathy or significant coronary artery atherosclerosis. Death was due to cardiomegaly. Peau d’orange was present of the lower abdominal wall with marked penile shortening and two testes present in the scrotum (Fig. 3).

### Discussion

Body weight, measured by BMI, has been increasing in many countries around the world for a number of years with an estimated rise of 0.4 kg/m^2^ per decade in a survey of 192 countries from 1980 to 2008 [1]. This has had a corresponding impact upon forensic mortuaries with increasing numbers of obese and morbidly obese decedents causing problems with the handling and transport of bodies, anatomical examinations and dissection, and the evaluation of the multitude of underlying associated comorbidities. Accelerated decomposition is another issue, as is subsequent disposal of such bodies as larger graves and crematoria may be required [2, 3].

Morbid obesity is associated with an increased risk of cardiovascular disease including ischemic heart disease and pulmonary thromboembolism, endocrine disease including diabetes mellitus, and gastrointestinal, metabolic, respiratory and infectious diseases. There is an increased incidence of a range of malignancies with greater risks of complications after surgery and during pregnancy [1].

**Fig. 4:**
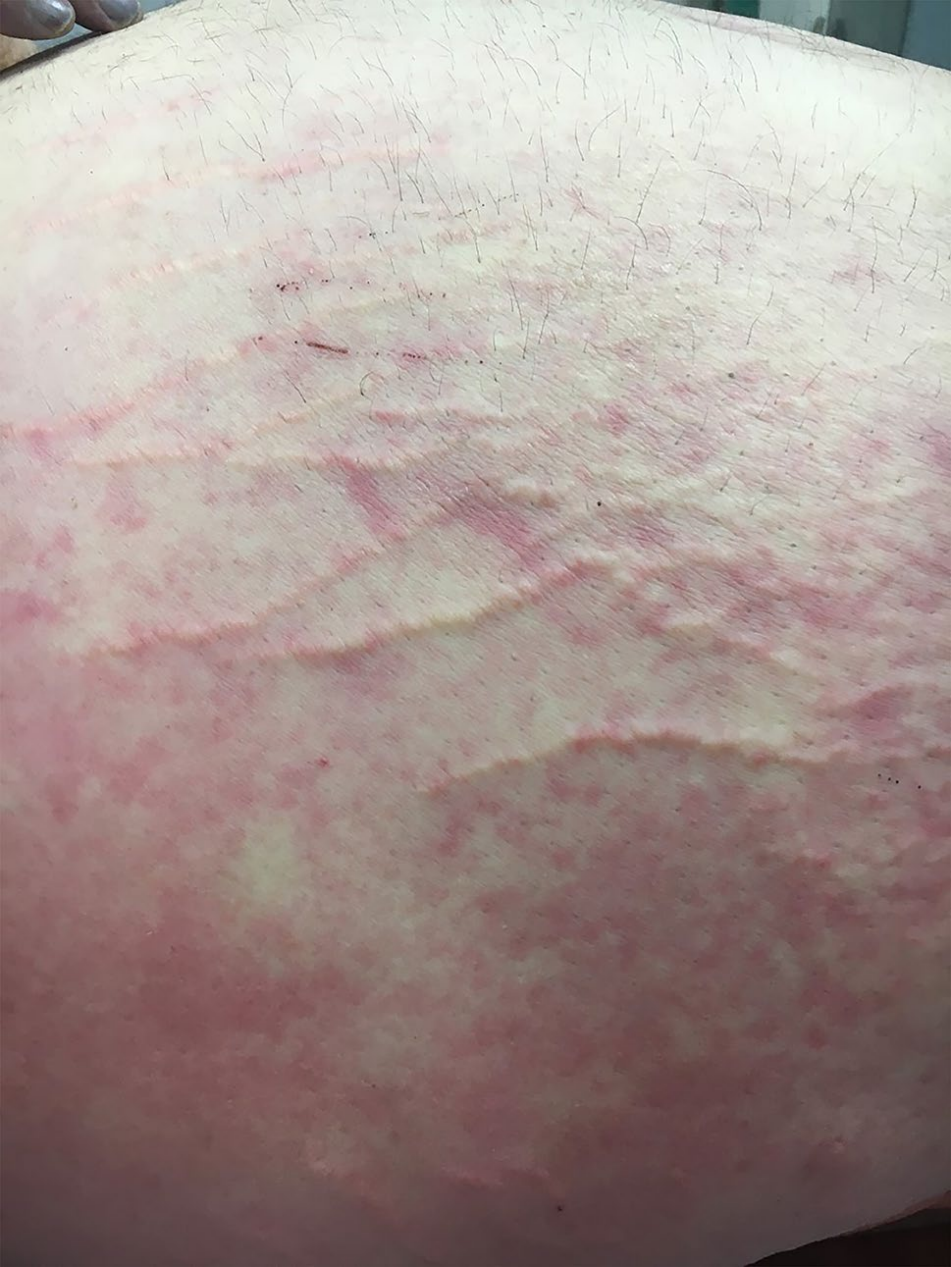
Peau d’orange changes in striae of the skin of the lower abdomen in a morbidly obese individual

**Fig. 5:**
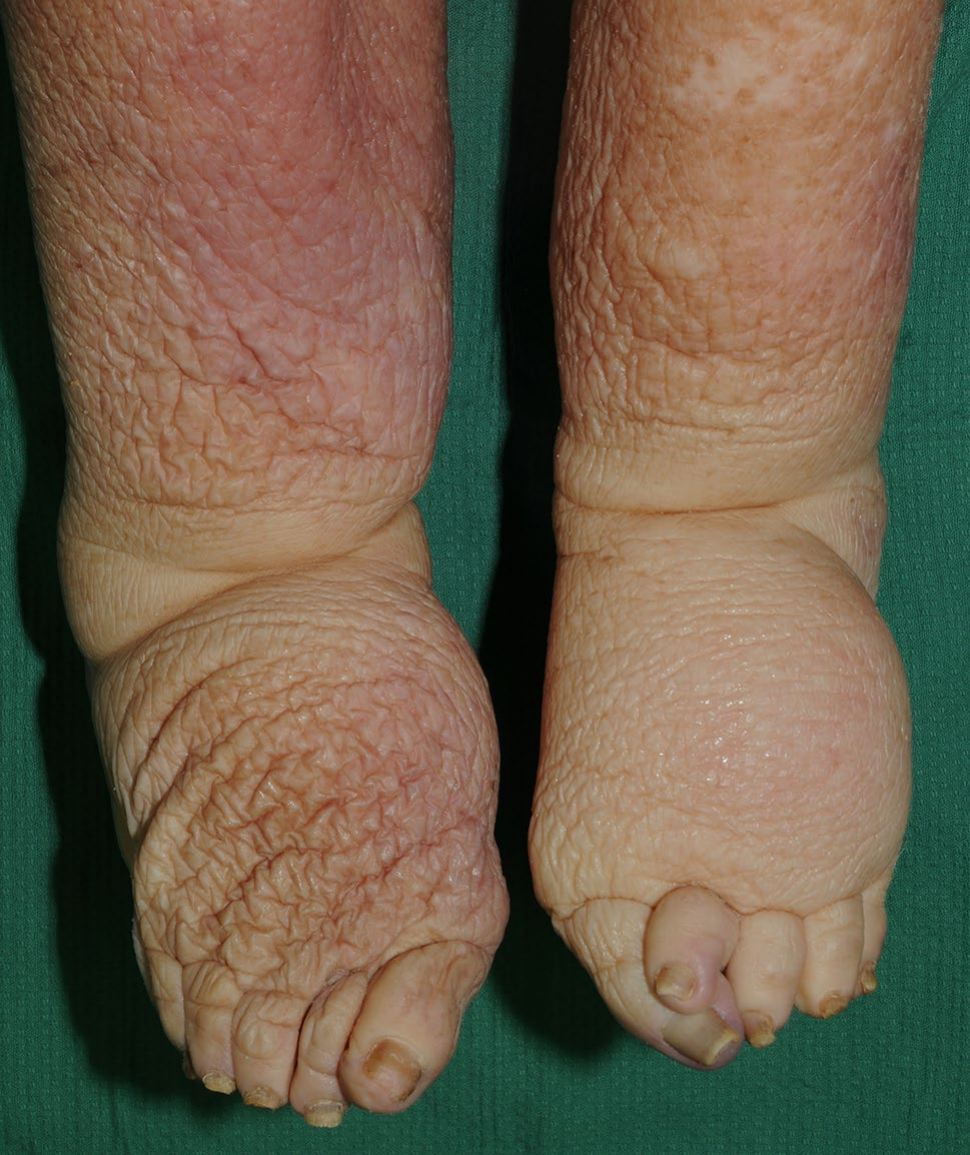
Lymphedema of the lower legs in a morbidly obese individual

A number of characteristic external features are present at the time of autopsy in morbidly obese decedents including abdominal distension with striae, skin tags, intertriginous rashes, peau d’orange changes of the skin [Fig. 4] and lymphedema [Fig. 5]. One feature that is not often commented upon in forensic cases is so-called burial or retraction of the penis. This was first described by Keyes in 1919 as “the apparent absence of the penis” [4]. It differs from a micropenis in that the penis is usually of normal length but has simply been enveloped by lower abdominal wall fat. This may result in considerable shortening of the externally displayed shaft such that the penis, as noted by Keyes, may appear to be completely missing [Fig. 3].

Although it may be congenital, one of the more common causes of a buried penis in adults is obesity associated with an overhanging suprapubic fat pad or escutcheon [5]. Other causes include scrotal lymphedema (from surgery or associated with elephantiasis due to nematodes such as *Wuchereria bancrofti*), scarring from circumcision or following penile/scrotal enlargement surgery, a dysgenic dartos muscle which does not provide adequate support for the penis, Fournier’s gangrene, lichen sclerosis and hidradenitis suppurativa [5].

Buried penis may be associated with local complications of bacterial and fungal infections due to chronic moistening of the area and issues with hygiene in the morbidly obese. Predisposing conditions may also lead to scarring and urethral stricture formation with urinary tract obstruction [6]. A study of 150 patients with adult acquired buried penis showed that 35% had premalignant changes with 7% having established invasive penile cancer [7]. This may also be influenced by obesity [8].

Concerns regarding the apparent shortening of a penis may not always be anatomically based, with *koro* a condition originally described in China and South East Asia, characterized by acute anxiety and panic with a belief that the penis is retracting or shrinking into the body [9]. Those who suffer from it complain of “genital hyperinvolution and fear of impending death” [10]. It has since been described in Europe, North America and Africa. Known also as shrinking penis and genital retraction syndromes, injuries may occur when ropes and equipment are attached to the penis in attempts to prevent it from reducing in size. An associated forensic issue occurs in parts of Africa where *koro* is thought to be associated with witchcraft and occult practices, resulting in those accused of penile theft sometimes being killed by angry mobs [11].

Although *koro* is a psychological condition with the diagnosis only being made if there are no underlying anatomical issues, it is unclear from the literature whether an initial reduction in penile length due to encroachment by accumulating suprapubic fat in obese individuals may contribute to fears of genital loss and initiate a more profound psychological reaction. While most episodes are usually solitary and self-limiting, [12], epidemics of *koro* continue to be reported occasionally in areas where health literacy is not robust. Such epidemics have been reported as recently as 2010 in India among migrant worker populations in labor camps [13]. It should also be noted that *koro* can also occur sporadically in individuals who fall outside the usual patient profile. For example, in 1997, a case was reported in the USA of a college-educated, overweight Caucasian male [14].

In conclusion, buried penis forms yet another albeit uncommon complication of morbid obesity which should initiate checking at autopsy for local infective and obstructive complications, with possible systemic effects. Histologic sampling of the area may identify underlying causative conditions or any malignant or premalignant changes. Predisposing conditions other than obesity should also be considered.
